# Assessment of the available therapeutic approaches for severe COVID-19: a meta-analysis of randomized controlled trials

**DOI:** 10.1038/s41598-023-44463-2

**Published:** 2023-10-10

**Authors:** Monika Marko, Rafał Pawliczak

**Affiliations:** Division of Biomedical Science, Department of Immunopathology, Faculty of Medicine, Medical University of Lodz, 7/9 Zeligowskiego St, 90-752 Lodz, Poland

**Keywords:** Diseases, Health care, Medical research

## Abstract

The study aimed to evaluate severe COVID-19 treatment approaches. We conducted a meta-analysis of randomized controlled trials (RTCs) with standard of care (SoC) as a control group and/or placebo. Database searching was performed separately for severe COVID-19 treatment such as anakinra, remdesivir, baricitinib, ivermectin, ritonavir, tocilizumab, sarilumab, sotrovimab, casirivimab/imdevimab. The results are presented as Risk Ratio (RR), 95% Confidence Interval (CI), and heterogeneity (I^2^). We obtained the most statistically significant outcomes favorable tocilizumab compared to SoC for death incidents RR 0.87 [95% CI 0.80, 0.95], overall effect p = 0.002, heterogeneity p = 0.85, I^2^ = 0%, need for mechanical ventilation RR 0.78 [95% CI 0.68, 0.89], overall effect p = 0.0004, heterogeneity p = 0.55, I^2^ = 0%, and number of patients discharged from hospital. RR 1.13 [95% CI 1.07, 1.20], overall effect p < 0.00001, heterogeneity p = 0.009, I^2^ = 85%. This meta-analysis has revealed that a considerable amount of research characterized by a very diverse methodology is available. Despite the limited data that met the criteria for inclusion in the meta-analysis, we showed that the available treatment options for severe COVID-19 are effective.

## Introduction

Severe acute respiratory syndrome coronavirus 2 (SARS-CoV-2) affected 626,090,018 people and caused the death of 6,564,556 confirmed worldwide (update on November 28, 2022)^1^. With reports on COVID-19 (coronavirus disease 2019) and its treatment options rising rapidly, there is a growing need to reassess and question treatment. COVID-19 has a broad clinical spectrum. Although most infected individuals experience only mild or subclinical disease, approximately 30% of hospitalized COVID-19 patients develop severe respiratory failure requiring intensive care^2^. According to a living World Health Organization (WHO) guideline on drugs for severe COVID-19^3^ corticosteroids, IL-6 receptor blockers (tocilizumab, sarilumab), and baricitinib (Janus kinase inhibitors) are strongly recommended in favor whereas remdesivir is conditionally recommended. Ruxolitinib and tofacitinib should be considered if neither baricitinib nor IL-6 receptor blockers are available. Conditional recommendations against severe COVID-19 include ivermectin (which can be used only in research settings) and remdesivir. Additionally, lopinavir/ritonavir, casirivimab imdevimab, and sotrovimab are strongly recommended against.

The decision on the selection of drugs included in the meta-analysis was based on the available reports on the treatment of COVID-19, including the WHO as mentioned above guidelines^3^. In the meta-analysis, it was decided to include drugs considered a therapy for mild COVID-19, such as monoclonal antibodies (mAbs) such as casirivimab and imdevimab. This decision was made based on available studies using these drugs for moderate to severe COVID-19. The European Medicines Agency also approves the premise of the combination of casirivimab and imdevimab for use in patients at high risk of progression to severe COVID-19 who do not require supplemental oxygen^4^.

Considering the newer and newer information reaching us and their updates, we decided to check whether we can assess what severe COVID-19 treatment is effective and what may fail us. To address this question, we conducted a meta-analysis of randomized controlled trials with standard of care (SoC) as a control group and/or placebo.

## Methods

### Search strategy

The databases PubMed, Cochrane Library and Clinical Trials (ct.gov.) were thoroughly searched, through October 2022. The search was performed in databases in 9 parts separately for Covid-19 treatment using these terms: 1. ((COVID-19) OR (coronavirus infection) OR (SARS-CoV-2) OR (coronavirus)) AND ((treatment) OR (therapy)) AND (anakinra), 2. ((COVID-19) OR (coronavirus infection) OR (SARS-CoV-2) OR (coronavirus)) AND ((treatment) OR (therapy)) AND (remdesivir), 3. ((COVID-19) OR (coronavirus infection) OR (SARS-CoV-2) OR (coronavirus)) AND ((treatment) OR (therapy)) AND (baricitinib), 4. ((COVID-19) OR (coronavirus infection) OR (SARS-CoV-2) OR (coronavirus)) AND ((treatment) OR (therapy)) AND (ivermectin), 5. ((COVID-19) OR (coronavirus infection) OR (SARS-CoV-2) OR (coronavirus)) AND ((treatment) OR (therapy)) AND (tocilizumab), 6. ((COVID-19) OR (coronavirus infection) OR (SARS-CoV-2) OR (coronavirus)) AND ((treatment) OR (therapy)) AND (sarilumab), 7. ((COVID-19) OR (coronavirus infection) OR (SARS-CoV-2) OR (coronavirus)) AND ((treatment) OR (therapy)) AND (ritonavir), 8. ((COVID-19) OR (coronavirus infection) OR (SARS-CoV-2) OR (coronavirus)) AND ((treatment) OR (therapy)) AND (sotrovimab), 9. ((COVID-19) OR (coronavirus infection) OR (SARS-CoV-2) OR (coronavirus)) AND ((treatment) OR (therapy)) AND (casirivimab), 10. ((COVID-19) OR (coronavirus infection) OR (SARS-CoV-2) OR (coronavirus)) AND ((treatment) OR (therapy)) AND (imdevimab). Database Clinical Trials (ct.gov) was searched with following criteria: completed studies, studies with results, interventional studies, randomized clinical trials, severe COVID-19.

Selecting drugs for the meta-analysis consisted of analyzing the available literature between September and October 2022. Two investigators performed the review independently using an internal record of text documents and spreadsheets. Then, the registered records were mutually evaluated and compared. The decision was made through discussion.

### Selection criteria

The research question and selection criteria were formulated using the Population, Intervention, Comparison, Outcomes, and Type of Study (PICOT) structure. The inclusion criteria were: (1) population: patients with severe COVID-19, (2) intervention: anakinra, remdesivir, baricitinib, ivermectin, tocilizumab, sarilumab, ritonavir, sotrovimab, casirivimab, imdevimab (3) comparison: placebo or standard of care (4) outcomes: must contain adverse events section, (5) must contain outcomes reported: mechanical ventilation use, hospitalization, discharge from hospital, death (6) type of study: clinical trial with randomization.

### Inclusion and exclusion criteria

The inclusion criteria were: (1) randomized clinical trials (RCTs). (2) Patients with severe COVID-19. (3) The intervention group: patients received treatment. The controlled group: patients received placebo or standard of care. (4) The results containing at least one of primary and secondary endpoint such as mechanical ventilation use, hospitalization, discharge from hospital, death, and adverse events.

The exclusion criteria were: (1) meta-analysis, (2) review article, (3) case series, (4) case report, (5) observational studies, (6) cohort studies, (7) animal research, (8) conference abstracts, (9) articles with insufficient information, (10) articles with insufficient data and (11) articles published in languages other than English.

### Assessment of the risk of bias and methodological quality

The methodological quality assessment was performed into the following classifications: low risk of bias, unclear risk of bias, and high risk of bias. Two investigators independently made an evaluation using the Cochrane Review Manager 5.4 software tool. The allowable value of losses that could affect the test results was set at 10% and final decisions were made in the discussion process in the case of opposing opinions of both investigators.

### Study selection and data extraction

Two authors independently evaluated the studies to minimize the potential risk of bias associated with article selection. The evaluation was based on predetermined inclusion and exclusion criteria. Attention was drawn to the possibility of duplicates or descriptions of the same results in different sources. Study dates and duration, authors' names, and study registration numbers were compared to eliminate these biases. In addition, the names of institutions' descriptions of interventions (frequency or dose) were checked, and the number of participants and results were compared. Both researchers cross-checked the selected data. All possible concerns were reported in a specially created file for each study. Then, the collected comments were discussed together, and in the absence of an agreement, negotiations were continued until a consensus was reached and a decision regarding the study was made.

It should also be emphasized that some studies do not use the term severity of the disease. However, from their context, it can be concluded that it is about the severe course of COVID-19. There are also reports on estimating the severity of the disease, suggesting that hospitalization is associated with a critical condition^5^. Therefore, it was decided that studies describing hospitalized patients would also be included.

The following information was extracted from included studies: title, authors, number and age of subjects, exposure and outcome, measures of exposure and research endpoints, and number of cases. Retrieved titles and abstracts were reviewed by the study authors. A full-text review was then performed on selected articles to confirm that inclusion and exclusion criteria were met. All randomized controlled trials that examined the effects of severe COVID-19 treatment—were considered eligible for inclusion. Studies that were assessed as eligible for meta-analysis against the PICOT criteria and quality were then screened for the availability of data that matched the meta-analytical calculations. Not all studies reported such data. Therefore, at the final stage of selection, many publications were rejected, resulting in a limited number of them in our meta-analysis.

### Statistical methods

The collected data was extracted and assembled in a standardized database using Cochrane Review Manager 5.4 software. A separate meta-analysis was performed for the trials with control as the standard of care and the placebo-controlled trials. Studies that compared the efficacy of more than one treatment approach versus placebo or control were presented and compared in the meta-analysis in two parts. The same scheme was used in case of different doses of the same treatment. We performed a meta-analysis stratified by treatment for AEs. The results comprised dichotomous data displayed as risk ratio (RR) and 95% confidence interval (CI) in each group.

Heterogeneity between study results was assessed using a standard chi-square test. I^2^ was used as a measure of heterogeneity. The following interpretation has been applied: I^2^ = 0–40% might not be important, I^2^ = 30–60% may be moderate, I^2^ = 50–90% may indicate substantial heterogeneity, and I^2^ = 75–100% indicated considerable heterogeneity. The results of this meta-analysis were considered statistically significant at P < 0.05.

### Ethics appproval

This article is a meta-analysis. It does not use any patients or personal data that could be submitted to the evaluation of an Ethics committee.

## Results

### Included studies

**Figure 1:**
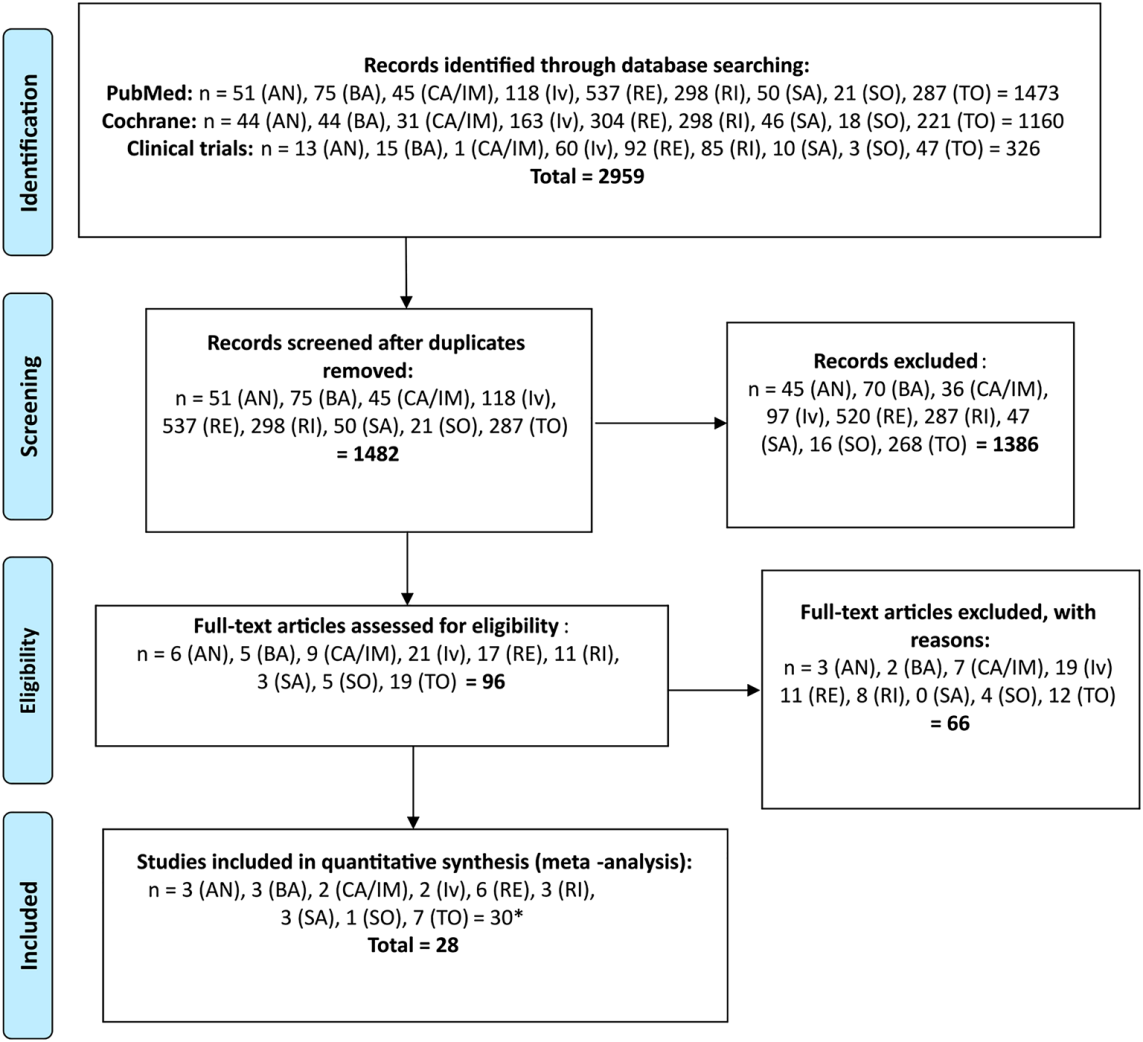
Flowchart of the screening procedure^6^. *Among the included studies, one study matched both the Tocilizumab and Anakinra, and one matched Tocilizumab and Sarilumab, ultimately resulting in the 28 included studies in the analysis. *AN* Anakinra, *BA* Baricitinib, *CA/IM* Casirivimab/Imdevimab, *Iv* Ivermectin, *RE* Remdesivir, *RI* Ritonavir, *SA* Sarilumab, *SO* Sotrovimab, *TO* Tocilizumab.

As shown in Fig. 1^6^ included 2959 related articles. In total, 1477 duplicates were removed. After our team's full‐text screening and quality evaluation, 28 articles meeting the inclusion criteria were included. Among the included articles, the following studies were used in meta-analysis: 3 for anakinra^7–10^, 3 for baricitinib^11–13^, 2 for casirivimab/imdevimab^4,14,15^, 2 for ivermectin^16,17^, 6 for remdesivir^18–23^, 3 for lopinavir/ritonavir^24–26^, 3 for sarilumab^27–29^, 1 for sotrovimab^12,29^, and 7 for tocilizumab^27,31–34^.

All the included studies were RCTs with a placebo or SoC as control conducted on patients suffering from severe COVID-19. Study details are shown in Table 1.

**Table 1:** Characteristics of included studies.

Study	Experimental group	Control/placebo group
Wang et al.^18^	Remdesivir	Placebo
Olender et al.^19^	Remdesivir	Standard of care
CMAJ^20^	Remdesivir	Standard of care
Beigel et al.^21^	Remdesivir	Placebo
Gottlieb et al.^22^	Remdesivir	Placebo
Mahajan et al.^23^	Remdesivir	Standard of care
Ader et al.^24^	Lopinavir/ritonavir	Standard of care
Arabi et al.^25^	Lopinavir–ritonavir	Standard of care
Cao et al.^26^	Lopinavir–ritonavir	Standard of care
Weinreich et al.^15^	REGEN-COV (casirivimab/imdevimab)	Placebo
RECOVERY Collabortive Group (1)^4^	Casirivimab/imdevimab	Standard of care
ACTIV-3^30^	Sotrovimab	Placebo
Kharazmi et al.^7^	Anakinra	Standard of care
Kyriazopoulou et al.^9^	Anakinra	Standard of care
Declercq et al.^10^	Anakinra, Tocilizumab	Standard of care
Hermine et al.^27^	Tocilizumab, Sarilumab	Standard of care
Merchante et al.^28^	Sarilumab (200 mg), Sarilumab (400 mg)	Standard of care
Lescure et al. (severe disease stratum)^29^	Sarilumab (400 mg) Sarilumab (200 mg)	Standard of care
Salvarani et al.^36^	Tocilizumab	Standard of care
RECOVERY Collaborative Group^31^	Tocilizumab	Standard of care
Soin et al.^32^	Tocilizumab	Standard of care
Rutgers et al.^33^	Tocilizumab	Standard of care
Broman et al.^34^	Tocilizumab	Standard of care
RECOVERY Collaborative Group (2)^11^	Baricitinib	Standard of care
Marconi et al.^12^	Baricitinib	Placebo
Ely et al.^13^	Baricitinib	Placebo
Reis et al.^16^	Ivermectin	Placebo
Vallejos et al.^17^	Ivermectin	Placebo

### Assessment of methodological quality

**Figure 2:**
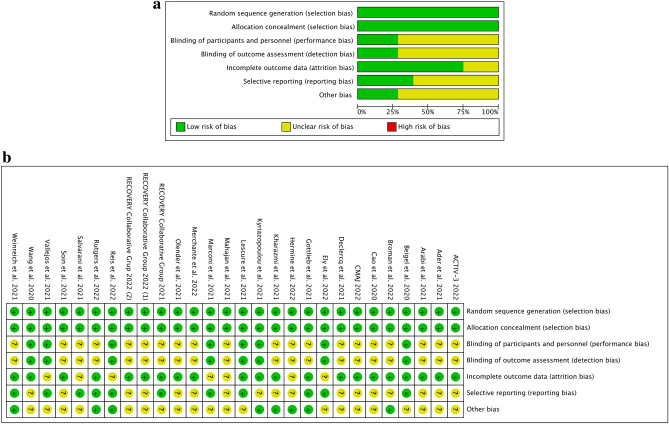
(**a**) Risk of bias graph: review authors' judgements about each risk of bias item presented as percentages across all included studies. (**b**) Risk of bias summary: review authors' judgements about each risk of bias item for each included study.

Twenty-eight studies were included in the risk of bias evaluation where the following item was assessed: random sequence generation (selection bias), allocation concealment (selection bias), blinding of participants and personnel (performance bias), blinding of outcome assessment (detection bias), incomplete outcome data (attrition bias), selective reporting (reporting bias) and other bias. Among the evaluated studies, no study had a high risk of bias in all the assessed items. All studies included in the meta-analysis were assigned a 100% low risk of selection bias (random sequence generation and allocation concealment). In contrast, more than 25% of the studies were rated low risk of blinding bias because they were described as double-blind. Approximately 75% of the studies were rated as low risk of incomplete data bias. About 40% of the studies were assessed as not at risk of selective reporting bias. On the other hand, more than 25% of studies were assessed as not endangered by other biases. The remaining percentage of studies were judged to have an unclear risk of bias in each of the parameters tested. The results obtained from the methodological evaluation are shown in Fig. 2a,b.

### Treatment effectiveness

**Figure 3:**
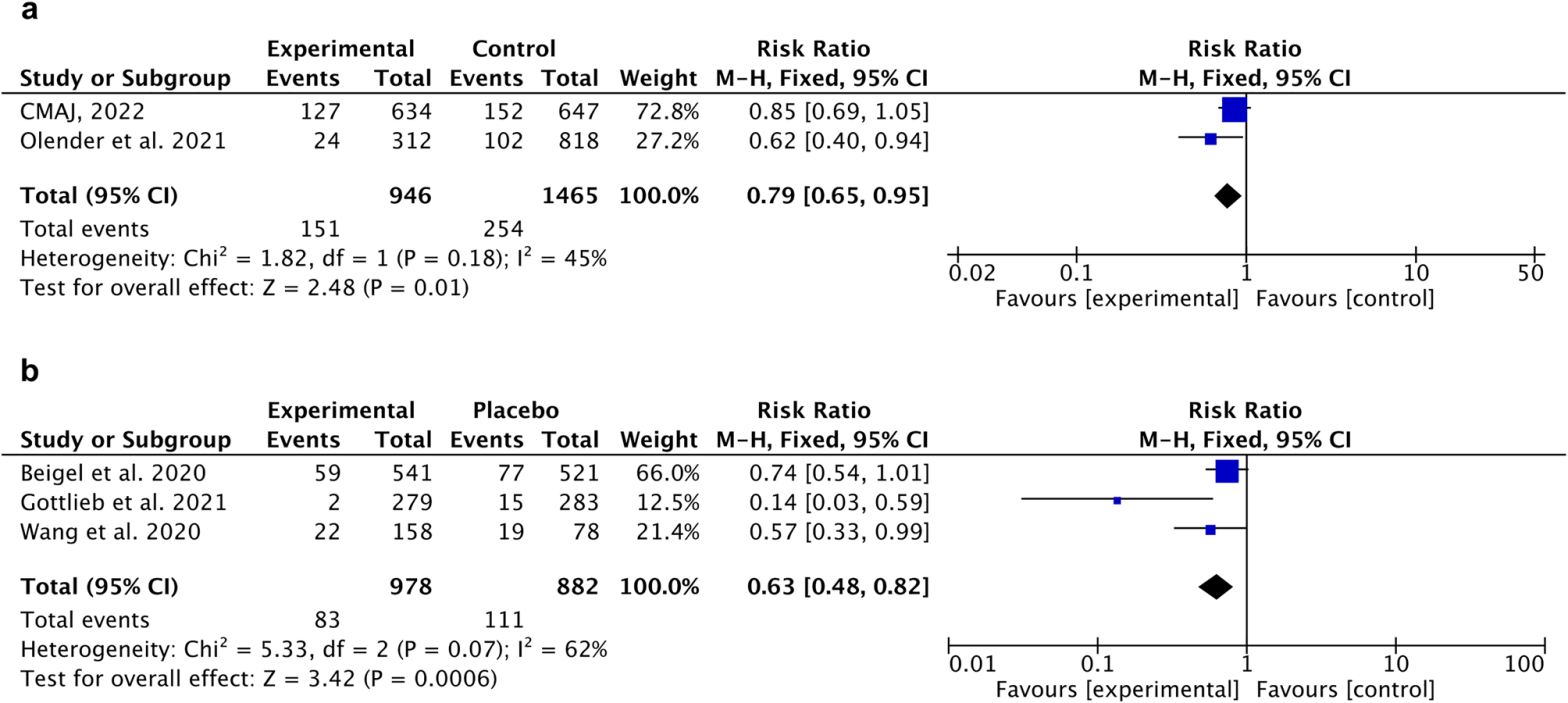
Forest plot for remdesivir. (**a**) Death incidence (compared to control), (**b**) Death incidence (compared to placebo).

For remdesivir, meta‐analysis demonstrated significantly less death incidence compared to control (SoC), RR 0.79 [95% CI 0.65, 0.95], overall effect p = 0.01, heterogeneity p = 0.18, I^2^ = 45% (Fig. 3a). The same trend was observed for remdesivir with placebo-controlled group, RR 0.63 [95% CI 0.48, 0.82], overall effect p = 0.0006, heterogeneity p = 0.07, I^2^ = 62% (Fig. 3b).

**Figure 4:**
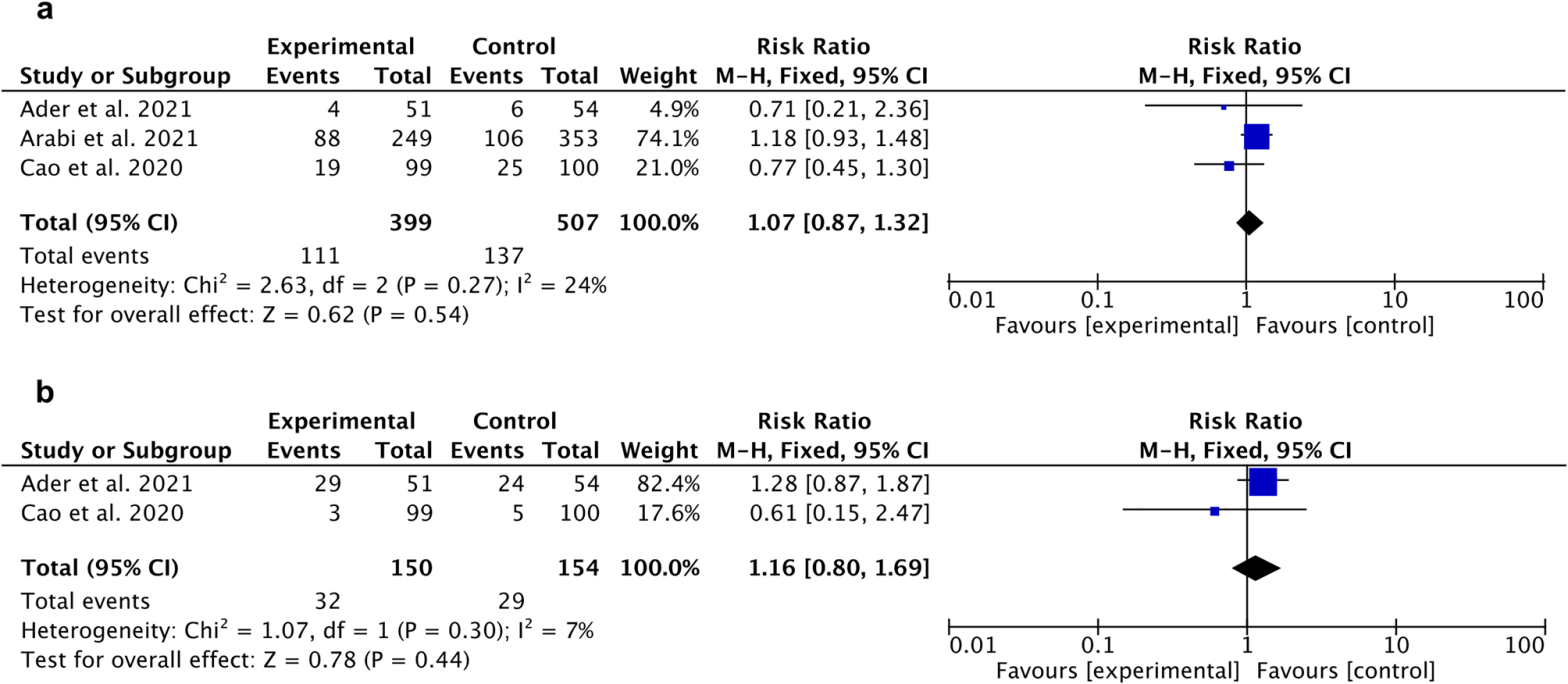
Forest plot for lopinavir/ritonavir. (**a**) Death incidence (compared to control), (**b**) Need for mechanical ventilation (compared to control).

For lopinavir/ritonavir compared to control (SoC), a meta-analysis showed no statistically significant outcomes for the control group in case of death incidence (Fig. 4a) and in a number of patients who needed mechanical ventilation (Fig. 4b).

**Figure 5:**
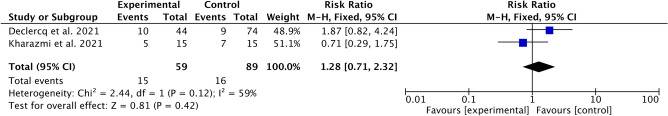
Forest plot for anakinra. Death incidence (compared to control).

**Figure 6:**
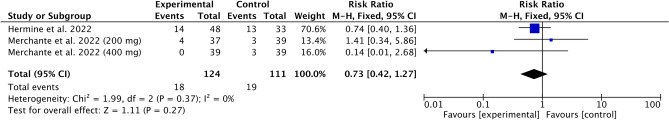
Forest plot for sarilumab. Death incidence (compared to control).

For anakinra (Fig. 5a) and sarilumab (Fig. 6) a meta‐analysis demonstrated no statistically significant outcomes for control (SoC) in case of death events (Figs. 5a and 6).

**Figure 7:**
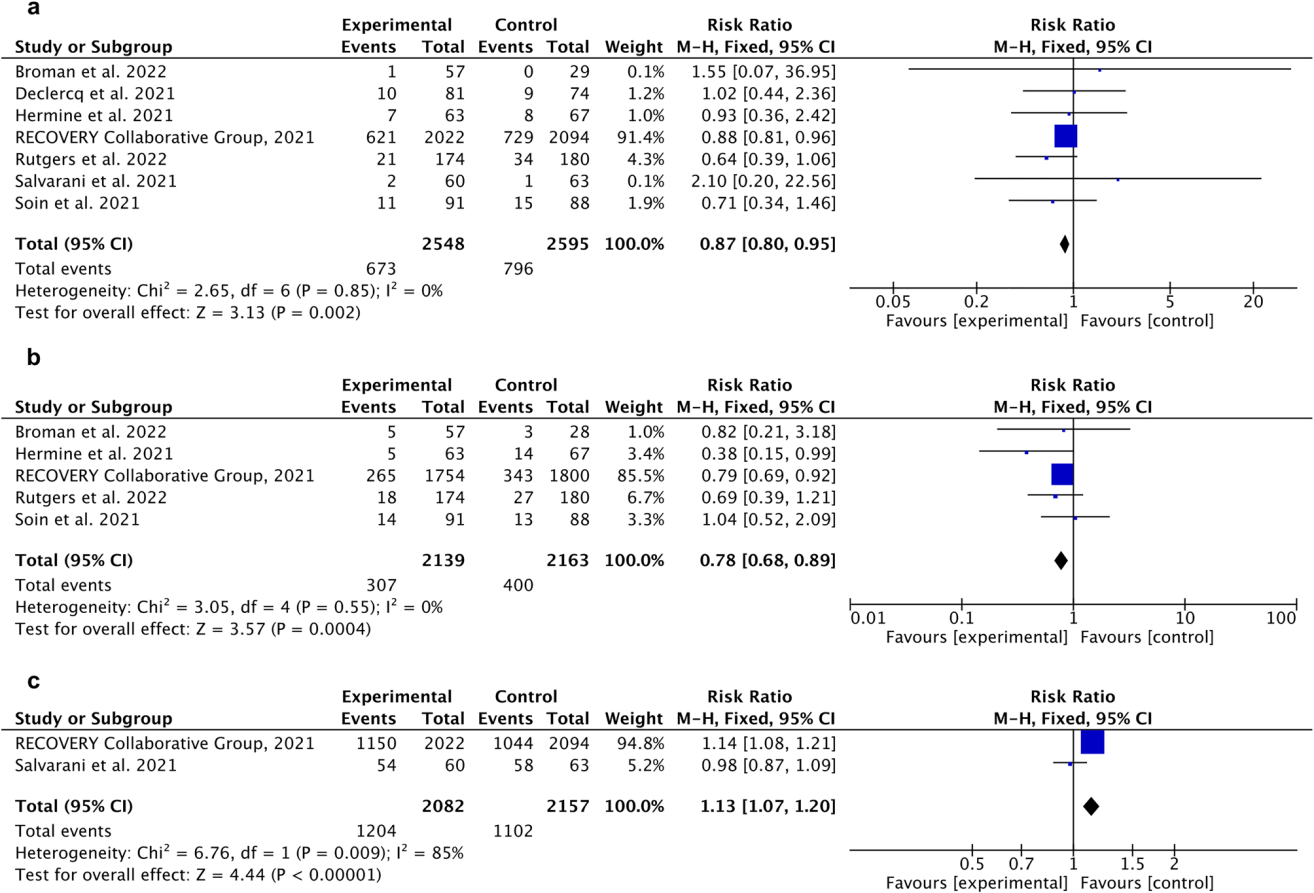
Forest plot for tocilizumab. (**a**) Death incidence (compared to control), (**b**) Need for mechanical ventilation (compared to control), (**c**) Patients discharged from hospital (compared to control).

For tocilizumab meta‐analysis revealed statistically significant less death events compared to control (SoC), RR 0.87 [95% CI 0.80, 0.95], overall effect p = 0.002, heterogeneity p = 0.85, I^2^ = 0% (Fig. 7a). The same trend was observed in case of need for mechanical ventilation with favorable results for experimental group RR 0.78 [95% CI 0.68, 0.89], overall effect p = 0.0004, heterogeneity p = 0.55, I^2^ = 0% (Fig. 7b). In addition, favorable results were observed for number of patients discharged from hospital RR 1.13 [95% CI 1.07, 1.20], overall effect p < 0.00001, heterogeneity p = 0.009, I^2^ = 85% (Fig. 7c).

**Figure 8:**
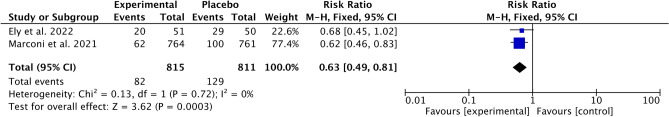
Forest plot for baricitinib. Death incidence (compared to placebo).

For baricitinib, we demonstrated significantly fewer death events than placebo, RR 0.63 [95% CI 0.49, 0.81], overall effect p = 0.0003, heterogeneity p = 0.72, I^2^ = 0% (Fig. 8).

**Figure 9:**
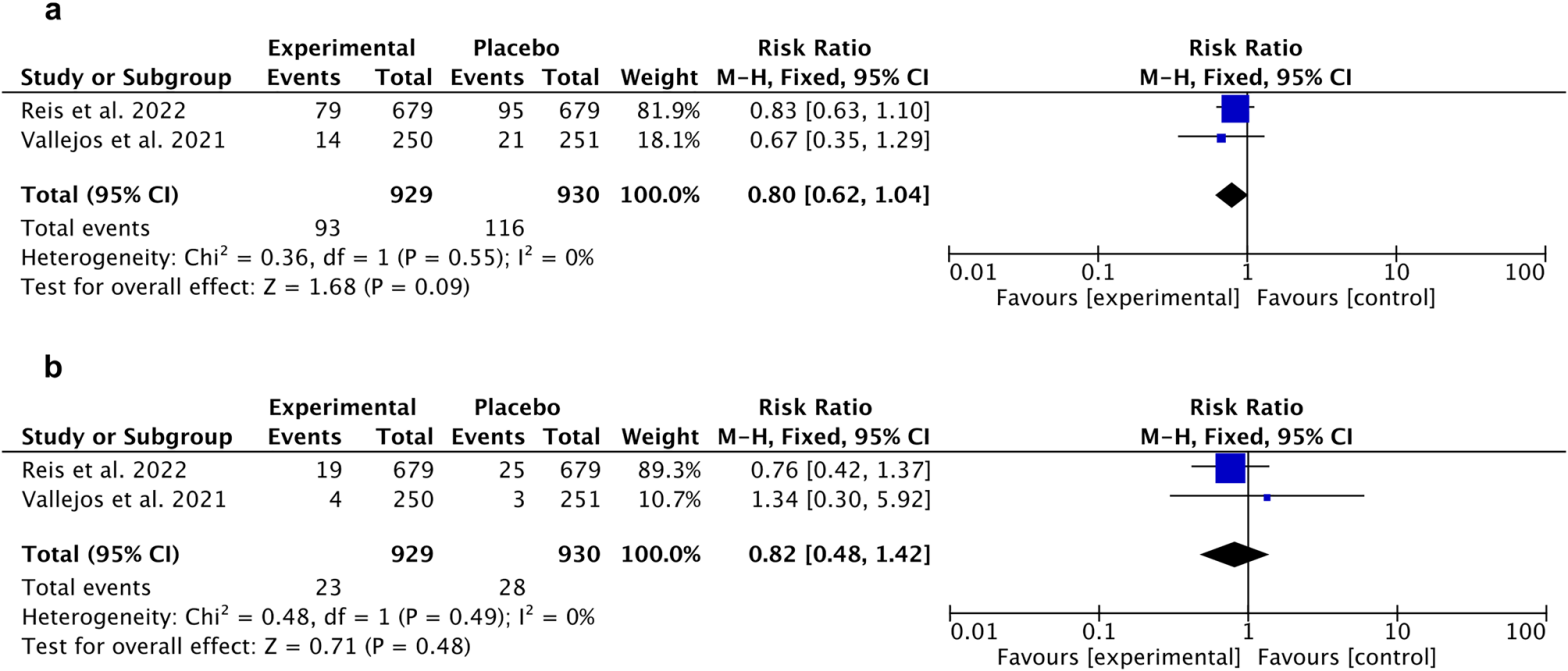
Forest plot for ivermectin. (**a**) Death incidence (compared to placebo), (**b**) Need for mechanical ventilation (compared to placebo).

For ivermectin, meta‐analysis demonstrated no statistically significant less hospitalized patients compared to placebo (Fig. 9a). Similarly, no statistically significant outcomes were obtained in an experimental group in the case of mechanical ventilation compared to placebo (Fig. 9b).

### Adverse events

**Figure 10:**
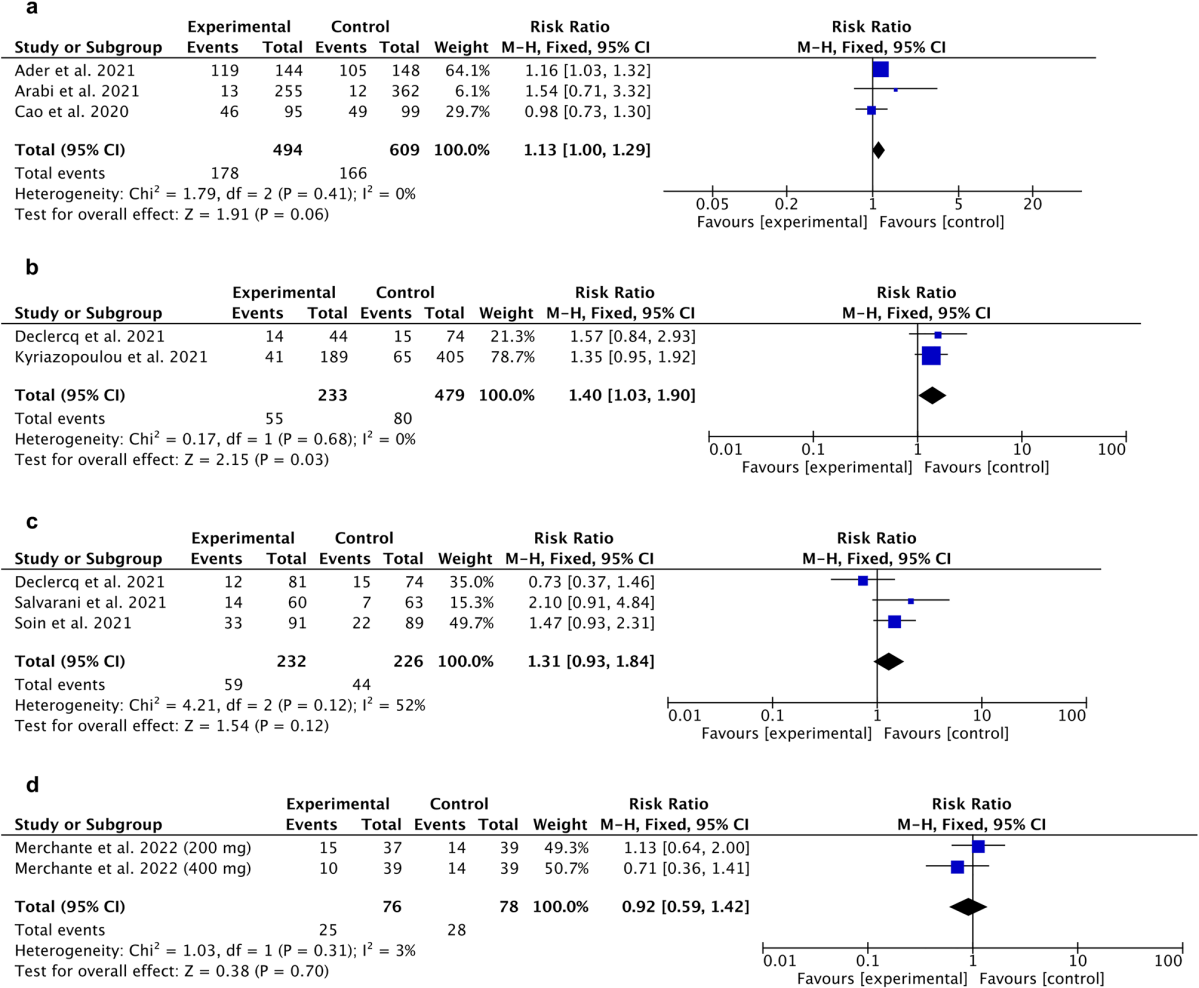
Forest plot for adverse events. Experimental compared to control. (**a**) Lopinavir/Ritonavir, (**b**) Anakinra, (**c**) Tocilizumab, (**d**) Sarilumab.

Meta-analysis of adverse events demonstrated statistically significant fewer AEs for anakinra RR 1.40 [95% CI 1.03, 1.90], overall effect p = 0.003, heterogeneity p = 0.68, I^2^ = 0% (Fig. 10b). For Lopinavir/Ritonavir (Fig. 10a), tocilizumab (Fig. 10c) and sarilumab (Fig. 10d) we obtain no statistically significant outcomes.

**Figure 11:**
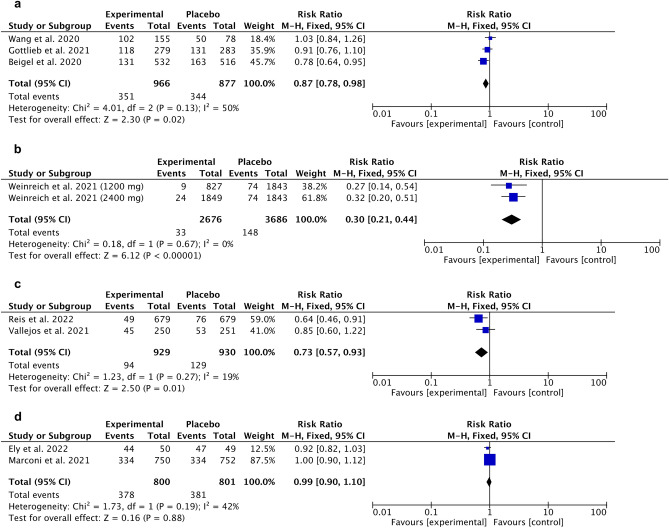
Forest plot for adverse events. Experimental compared to placebo. (**a**) Remdesivir, (**b**) Casirivimab/Imdevimab, (**c**) Ivermectin, (**d**) Baricitinib.

Opposed results were obtained in selected studies where the experimental group was compared to the placebo. Favorable outcomes were observed for remdesivir RR 0.87 [95% CI 0.78, 0.978], overall effect p = 0.02, heterogeneity p = 0.13, I^2^ = 50% (Fig. 11a), casirivimab/imdevimab RR 0.30 [95% CI 0.21, 0.44], overall effect p < 0.00001, heterogeneity p = 0.67, I^2^ = 0% (Fig. 11b), ivermectin RR 0.73 [95% CI 0.57, 0.93], overall effect p = 0.01, heterogeneity p = 0.27, I^2^ = 19% (Fig. 11c). For baricitinib we obtained no statistically significant outcomes (Fig. 11d).

## Discussion

The present meta-analysis shows that treatment approaches compared in the study, in most cases, have a positive effect in treating severe COVID-19. We demonstrated the statistically significant mortality benefits with the use of remdesivir (both in the comparison to SoC and placebo, tocilizumab (compared to SoC), and baricitinib (compared to placebo).

Our data showed that patients have achieved positive results in reducing the need for mechanical ventilation for tocilizumab (compared to SoC). In our study, we investigated the influence of the studied drugs on the frequency of discharging patients from the hospital after their use. After a literature review and selection, only two studies for tocilizumab were included in this comparison, which gives limited results. Patients treated with tocilizumab noticed more cases discharged from the hospital compared to the group with SoC. These results suggest that this treatment option prevents hospitalization in patients with severe COVID-19.

There is little possibility of comparing the results of our meta-analysis to the already existing publications due to the need for more consistency in the methodology. This may be related to the considerable amount of incoming data on COVID-19 treatment and a vast field of research and observations on many disease entities. This could be the reason for the high risk of bias between the studies.

Adverse events were also analyzed for the trials referring to SoC as a control and the placebo-controlled trials. Our results show that statistically, significantly fewer AEs were found for SoC compared to anakinra. Additionally, significantly fewer AEs were observed in remdesivir, casirivimab/imdevimab, and ivermectin groups compared to placebo.

It should also be noted that in the meta-analytical calculations, we obtained wide confidence intervals in some cases, which affects the clinical significance of the result. This included cases such as death incidence for anakinra and sarilumab (compared to control), the need for mechanical ventilation for ivermectin (compared to placebo), and adverse events for tocilizumab and sarilumab (compared to control). We did not obtain statistically significant results in these cases, along with the wide confidence interval.

Our results from AEs meta-analysis can be applied to previously published meta-analyzes that also confirmed the occurrence of AEs during COVID-19 treatment^35^. However, caution should be exercised in analyzing these data because many meta-analyzes of AEs incidence during COVID-19 treatment do not accurately define whether the authors considered the severity of the disease, which in turn is essential in this work.

Since the beginning of the SARS-CoV-2 pandemic, many studies and meta-analyses have appeared on various therapeutic aspects used in the course of COVID-19 infection. As practitioners and scientists, we are faced with the challenge of verifying the newer and newer information and reports reaching us, which raises many questions. There is an ever-increasing need to expand and update knowledge about available treatment options. We need to know which of them are effective and safe. In our meta-analysis, we were able to analyze eight therapeutic options for COVID-19, including remdesivir, lopinavir/ritonavir, casirivimab/imdevimab, anakinra, sarilumab, tocilizumab, baricitinib and ivermectin. It should be emphasized that we were unable to use some of the included studies in meta-analytical comparisons. Due to the inability to compare it with other studies and the lack of relevant data, the Recovery 2022 (1)^4^ and Recovery 2022 (2)^11^ studies for casirivimab/imdevimab and baricitinib were not used in the meta-analysis.

As a result of the literature review, several clinical studies related to treating COVID-19 infection and its consequences have emerged and received widespread attention. However, complete data from randomized controlled trials still need to be included. Therefore, our meta-analysis has some limitations that should be considered when analyzing its results.

There is much research on the therapeutic options of interest to us that we wanted to analyze in our study. However, a review and screening of the literature showed that only some still need to be applied after applying our criteria for inclusion in the meta-analysis. We aspired the research included in the meta-analysis to be consistent and relevant. We searched for studies with a well-defined course of COVID-19. In our case, it was supposed to be a severe course. A large amount of research at this selection stage was excluded because they needed more information on the severity of the disease or were mainly concerned with mild or moderate disease. Next, we wanted to include randomized trials. We found studies that looked at either a placebo or a SoC control. To provide more data in our meta-analysis, separate comparisons were used for both placebo and controls.

## Conclusion

Considering the statistical aspect of our study, due to the statistical significance and heterogeneity values, tocilizumab was the best given the number of deaths, number of patients needing mechanical ventilation, and number of patients discharged from the hospital compared to SoC. It should be noted that the most significant number of comparisons (for several endpoints) were made for remdesivir, anakinra, and tocilizumab, which proves that the studies on them are the most consistent. The results relate to WHO recommendations^3^ for severe COVID-19, where tocilizumab is recommended. This meta-analysis has revealed many COVID-19 studies characterized by a very different methodology. Therefore, the results of this study should be interpreted with caution. Despite the limited data that met the criteria for inclusion in the meta-analysis, we showed that the available treatment options for severe COVID-19 are effective and can be safely used.

## Data Availability

All data generated or analysed during this study are included in this published article.
